# Associations Between Perceived Fatigability and Amyloid Status in the Baltimore Longitudinal Study of Aging

**DOI:** 10.1093/geroni/igab046.799

**Published:** 2021-12-17

**Authors:** Fangyu Liu, Ryan Dougherty, Amal Wanigatunga, Murat Bilgel, Yang An, Eleanor Simonsick, Susan Resnick, Jennifer Schrack

**Affiliations:** 1 Johns Hopkins University, Baltimore, Maryland, United States; 2 Johns Hopkins Bloomberg School of Public Health, Baltimore, Maryland, United States; 3 National Institute on Aging, Baltimore, Maryland, United States; 4 NIA, Baltimore, Maryland, United States; 5 National Instute on Aging/NIH, Baltimore, Maryland, United States

## Abstract

Higher level of and greater longitudinal increase in perceived fatigability are linked to cognitive decline and lower brain volumes in older adults. However, it remains unclear whether perceived fatigability is associated with Alzheimer’s disease-related brain pathology. In the BLSA, 163 participants without neurological disease or cognitive impairment (aged 74.7+/-8.4 years, 45% men) were assessed for perceived fatigability using rating of perceived exertion after a 5-minute (0.67 m/s) treadmill walk and Aß burden using 11C-Pittsburgh compound B (PiB) positron emission tomography. Forty-four participants were PiB+ based on a mean cortical distribution volume ratio (DVR) cut point of 1.066. After adjusting for demographics, body composition, comorbidities and ApoE-e4, higher perceived fatigability was not associated with PiB+ status (OR=0.84; 95% CI: 0.69, 1.05). Results suggest perceived fatigability may contribute to cognitive decline through pathways other than Aß pathology. Future studies should target other mechanisms linking perceived fatigability and cognitive decline.

